# Decreased expression of FBXW7 by ERK1/2 activation in drug-resistant cancer cells confers transcriptional activation of MDR1 by suppression of ubiquitin degradation of HSF1

**DOI:** 10.1038/s41419-020-2600-3

**Published:** 2020-05-26

**Authors:** Gil-Im Mun, Eun Choi, Yeongmin Lee, Yun-Sil Lee

**Affiliations:** Graduate School of Pharmaceutical Sciences, Ewha Womans University, Seoul, 120-750 Korea

**Keywords:** Cancer therapeutic resistance, Phosphorylation

## Abstract

The acquisition of MDR1-mediated chemoresistance poses a major obstacle to the success of conventional chemotherapeutic agents. HSF1 is also involved in chemoresistance, and several studies have demonstrated the relationship between HSF1 and MDR1 but without any consistent results. Paclitaxel- and doxorubicin-resistant cancer cells showed higher expression of MDR1 and HSF1. Depletion of HSF1 decreased *mdr1* expression at mRNA level, and HSF1 directly interacted with the promoter site of *mdr1*, suggesting its role as a transcriptional regulator of MDR1. Phosphorylation of Ser303/307, which was involved in protein stability of HSF1 by FBXW7-mediated degradation, was found to be important for transcriptional activation of *mdr1*. Drug-resistant cells showed decreased expression of FBXW7, which was mediated by the activation of ERK1/2, thus indicating that over-activation of ERK1/2 in drug-resistant cells decreased FBXW7 protein stability, which finally inhibited protein degradation of pHSF1 at Ser303/307. There was a positive correlation between immunofluorescence data of pHSF1 at Ser303/307 and MDR1 in carcinogen-induced rat mammary tumors and human lung cancers. These findings identified the post-translational mechanisms of HSF1 transcription in MDR1 regulation of drug resistance development.

## Introduction

Heat-shock factor 1 (HSF1) is a master regulator of the heat-shock response and facilitates cell survival and proliferation in eukaryotes. It has been widely reported that HSF1 is often overexpressed in cancer cells, thus suggesting that it has a role in tumorigenesis. HSF1 is found to mediate the protection of cancer cells from programed cell death by overriding cell cycle checkpoints and thus exacerbating metastasis. Activation of HSF1-dependent stress response, a cytoprotective mechanism, may greatly influence the development of an adaptive and protective phenotype in cancer cells subjected to anticancer agents. Elevated expression of heat-shock proteins (HSPs) has been reported in many types of human malignancies and is reportedly associated with resistance of cancer cells to apoptosis induced by chemotherapeutic agents^1–3^. In addition, HSP-independent mechanisms are also reportedly involved in HSF1-regulated resistance of cancer cells to chemotherapeutics^4,5^.

Post-translational modifications have fundamental roles in the activation and suppression of HSF1, including subcellular localization of HSF1 and interaction of HSF1 with partner proteins. Although the specific requirements for phosphorylation events and their specific roles in the regulation of HSF1 remain unclear, 12 phosphorylated serine residues have been identified^6^. Regarding phosphorylation, several serine residues appear to participate in the regulation of HSF1 transcription activity. Among these, phosphorylation of Ser230, Ser320, Ser326, and Ser419 contributes to activation of HSF1, whereas phosphorylation of Ser121, Ser303, Ser307, and Ser363 represses its activity^7^.

Previous studies have suggested that HSF1 activation is associated with poor outcome in breast cancer^8^, and reduced FBXW7 expression or inactivating mutations is significantly correlated with poor patient prognosis in multiple cancers^9^. HSF1 accumulation due to altered expression of a substrate-targeting subunit of the SCF (Skp-1-Cull-F-box) ubiquitin ligase complex, FBXW7, provides an advantage in cancer cells during disease progression. FBXW7 targets several key regulators of proliferation, growth, and apoptosis for proteasomal degradation^10^, and FBXW7 is mutated in a significant portion of diverse human cancers^11–13^. The interaction between FBXW7 and HSF1 is reported and FBXW7 controls the stability of nuclear HSF1^14^.

The acquisition of the multidrug-resistance (MDR) phonotype, defined as increased resistance against cytotoxic drugs with unrelated structures, represents one of the major obstacles for chemotherapy of tumors and other malignancies. The MDR gene was involved in MDR phenotype^15^. Drug resistance is acquired by prolonged exposure to cytotoxic drugs with the amplification of the *mdr1* gene. The transcriptional regulation of *mdr1* is tissue specifically induced; however, the molecular mechanisms are not yet fully clarified. HSF1 can participate in *mdr1* gene expression. Heat-shock elements (HSE) have been identified in the *mdr1* gene promoter^16,17^, and typical stress inducers, such as heat shock and arsenite, which induce HSP gene expression, also induce *mdr1* gene expression in some cell types^16,17^. Some MDR cell lines exhibit constitutively high HSF1-DNA binding activity^18^, and quercetin can inhibit the HSF1–HSE binding and *mdr1* gene expression in MDR cells^19^. However, some reports suggest that the activation of MDR expression by heat shock and other stressors may be mediated by DNA sequences and transcription factors besides HSE of HSF1 (refs. ^20–22^).

Several reports have demonstrated the relationship between HSF1 and MDR1. However, the precise role of HSF1 on the expression of MDR1 remains unclear. Several studies have presented the evidence that HSF1 is often overexpressed in chemoresistant cancer cells and that it upregulates the transcription of *mdr1*, thereby enhancing the efflux of drugs^4^. Conversely, other reports have suggested an interplay between HSF1 and nuclear factor κB (NFκB)^23^. Studies have shown that HSF1 suppresses NFκB activity by interrupting its nuclear binding on DNA. Silencing HSF1 may lead to higher NFκB expression and activity and may enhance antiapoptotic activity in cells^24^.

Paclitaxel is used in platinum-based doublet regimens as the first-line standard therapy for advanced NSCLC^25^. Despite its widespread use, its clinical effectiveness is limited by the development of paclitaxel-resistant cancer cells, which eventually leads to poor prognosis and relapse^26,27^. Various mechanisms involved in acquired paclitaxel resistance have been reported. The best understood mechanism of paclitaxel resistance involves the overexpression of MDR1, which is encoded by the *mdr1* gene conferring the multidrug-resistance phenotype^28^. However, further understanding of precise mechanisms involved in paclitaxel resistance is greatly warranted.

In this study, chemotherapeutic agent (paclitaxel or doxorubicin)-resistant cancer cells showed high expression of MDR1 and increased protein stability of HSF1, which were related to the paclitaxel-mediated resistance. Moreover, the phosphorylation of HSF1 at Ser303/307, which controlled HSF1 protein stability by FBXW7-mediated ubiquitin degradation, was involved in transcriptional activation of *mdr1*, which may affect drug resistance.

## Results

### Increased expression of HSF1 after paclitaxel treatment in drug-resistant cancer cells accompanied with transcriptional activation of the *mdr1* gene

**Fig. 1:**
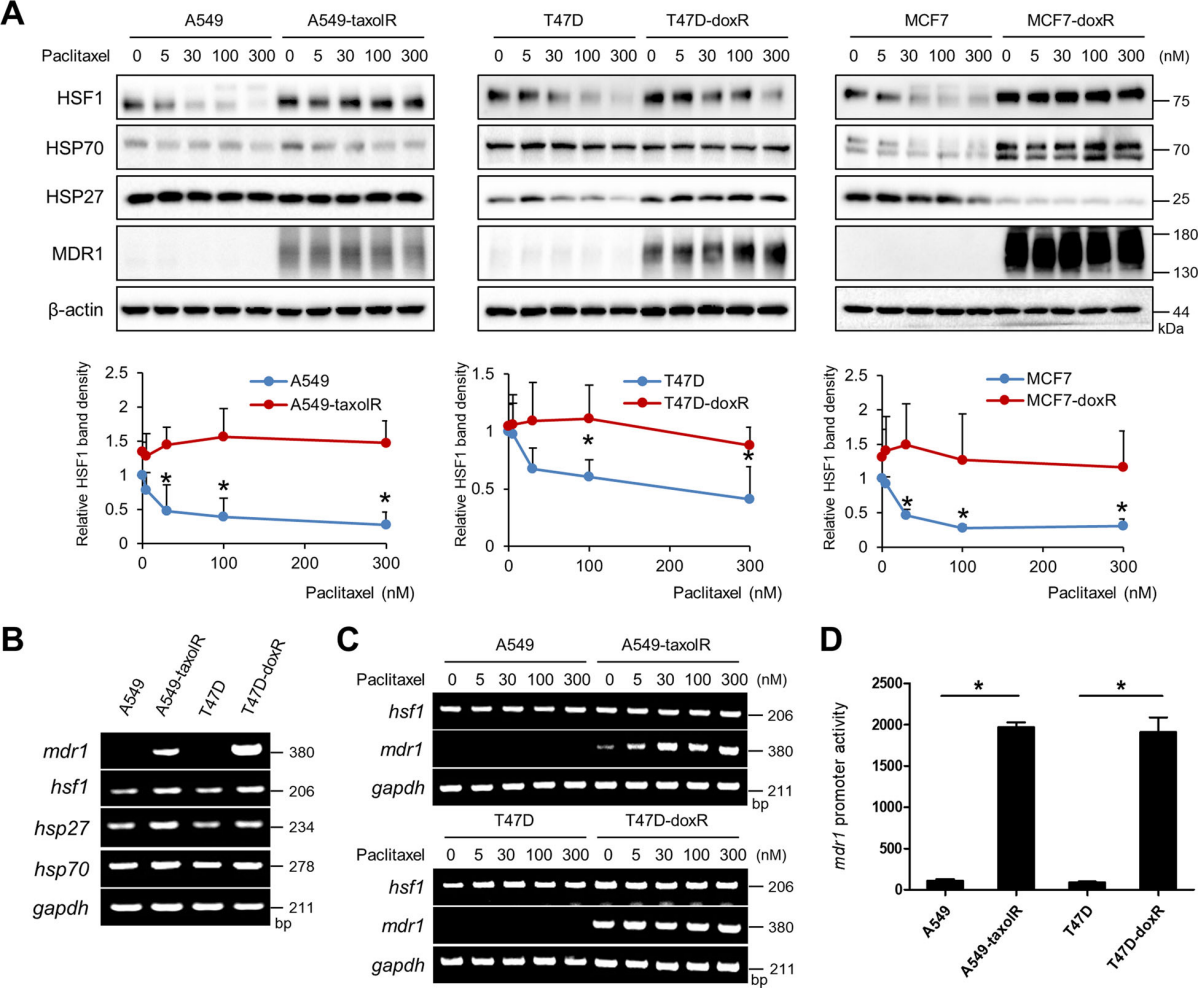
The expression of HSF1 and MDR1 was up-regulated in drug-resistant cancer cells. Western blotting (**a**) or RT-PCR (**b**, **c**) using A549 lung cancer cells, paclitaxel-resistant A549 cells (A549-taxolR), T47D breast cancer cells, doxorubicin-resistant T47D cells (T47D-doxR), MCF7 breast adenocarcinoma cells, and doxorubicin-resistant MCF7 cells (MCF7-doxR) was performed with or without treatment with paclitaxel at indicated concentrations for 24 h; *gapdh* was used as a loading control for RT-PCR. Band density was expressed as the fold change relative to the control in graphs. **d** Luciferase assays were performed after transfection with a luciferase reporter construct with the *mdr1* promoter. Values are expressed as fold change relative to the negative control and presented as the mean ± SD of at least three independent experiments. Statistics calculated based on a Student’s *t*-test or one-way ANOVA, *p < 0.05 vs each parent cell line.

To elucidate the involvement of HSF1 in drug resistance, paclitaxel-resistant A549 lung cancer cells (A549-taxolR) were generated by sustained treatment with 100-nM paclitaxel to maintain the paclitaxel resistance phenotype^29^. In the case of doxorubicin (T47D-doxR or MCF7-doxR)-resistant T47D and MCF7 cells, they were previously reported to be resistant to doxorubicin^30,31^. All the resistance cells of A549-taxolR, T47D-doxR, and MCF7-doxR showed resistance to paclitaxel treatment on caspase-3 or PARP1 cleavage detection and cell viability assays. IC_50_ values after paclitaxel treatment were 4.4 ± 0.15 μM for A549, 0.77 ± 0.08 μM for T47D, and 0.73 ± 0.03 μM for MCF7 cells (MTT assay after 24 h treatment of paclitaxel). The degree of resistance in drug-resistant cells after paclitaxel treatment was 23.3% for A549-taxolR, 29.9% for T47D-doxR, and 32% for MCF7-doxR. A549-taxolR was less sensitive to paclitaxel than T47D-doxR or MCF7-doxR (Supplementary Fig. 1). These resistant cells showed increased expression of HSF1 and MDR1, which confers the MDR phenotype. Moreover, increasing dose of paclitaxel treatment did not affect HSF1 expression in drug-resistant cells, whereas HSF1 expression after paclitaxel treatment was dose-dependently inhibited in control cells. MDR1 expression was the highest in MCF7-doxR cells and the lowest in A549-taxolR cells (Fig. 1a). Reverse transcriptase PCR (RT-PCR) data revealed that the *mdr1* gene was overexpressed in both A549-taxolR and T47D-doxR cells; the *hsf1* gene levels were not changed (Fig. 1b). Paclitaxel treatment affected mRNA of *mdr1* more in drug-resistant cells (Fig. 1c). Promoter activity of *mdr1* was increased in both A549-taxolR and T47D-doxR cells when compared with their parent cells (Fig. 1d), suggesting that chemotherapeutic drug-resistant cells showed increased expression of MDR1 and HSF1; MDR1 expression was regulated at a transcriptional level and HSF1 expression at a post-translational level.

**Fig. 2:**
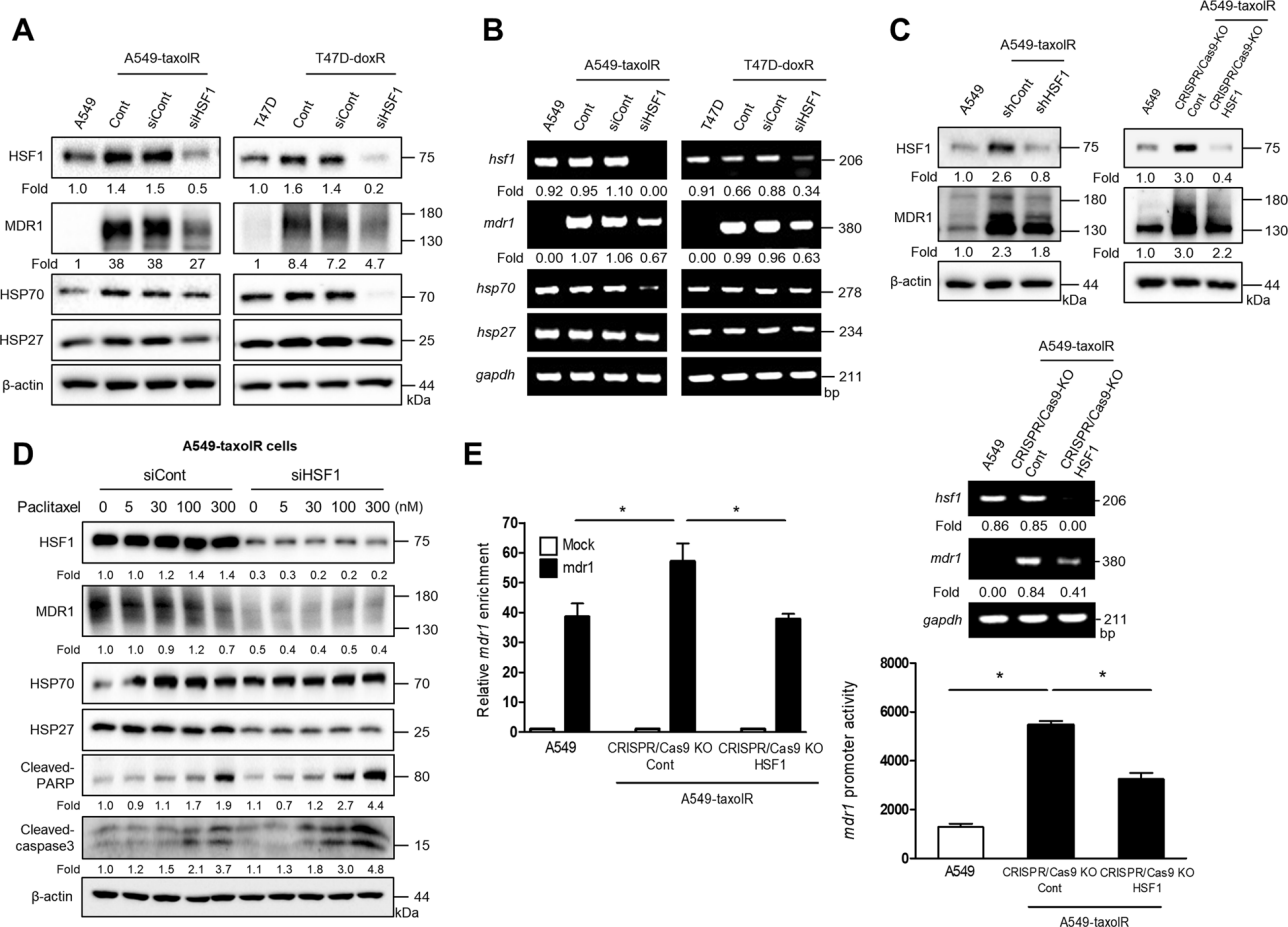
HSF1 depletion down-regulated the transcriptional level of *mdr1* in drug-resistant cancer cells. **a, b** After A549 lung cancer cells, paclitaxel-resistant A549 cells (A549-taxolR), T47D breast cancer cells, and doxorubicin-resistant T47D cells (T47D-doxR) were transfected with a control siRNA (siCont) or siHSF1, western blotting or RT-PCR was performed. **c** After A549 and A549-taxolR were transfected with shRNA (shCont), shHSF1, CRISPR/Cas9-Control (Cont), or HSF1 CRISPR/Cas9 knockout (KO) plasmid, western blotting (top) or RT-PCR (middle) was performed. **c**, bottom Luciferase assays in A549 and A549-taxolR with CRISPR/Cas9-Control or HSF1 CRISPR/Cas9 KO plasmid were performed after transfection with a luciferase reporter construct with the *mdr1* promoter. Values are presented as the mean ± SD of at least three independent experiments. Statistics calculated based on one-way ANOVA, *p < 0.05. **d** A549-taxolR cells were treated with indicated concentrations of paclitaxel for 24 h after transfection with siCont or siHSF1, and western blotting was performed. **e** A549 and A549-taxolR with CRISPR/Cas9-Control or HSF1 CRISPR/Cas9 KO plasmid were analyzed by ChIP assay to measure enrichment of *mdr1* promoter sequences. IgG was used as a negative control for the HSF1 antibody. Statistics calculated based on one-way ANOVA, *p < 0.05. Values are expressed as fold change relative to the control.

To identify the possible relationship between HSF1 and MDR1, HSF1 was knockdowned to A549-taxolR and T47D-doxR cells using siRNA directed against HSF1 (siHSF1); this led to down-regulation of MDR1 at both protein and mRNA levels (Fig. 2a, b). Knockdown of *hsf1* by the CRISPR/Cas9 system or stable transfection of shRNA to A549-taxolR cells also showed decreased levels of both MDR1 protein and mRNA as well as *mdr1* promoter activity (Fig. 2c, Supplementary Fig. 2a). Paclitaxel treatment to siHSF1-transfected A549-taxolR cells showed decreased expression of MDR1 and increased apoptosis when compared with control siRNA-treated cells (Fig. 2d). Chromatin immunoprecipitation (Chip) assay revealed that HSF1 was more enriched in the promoter region of *mdr1* in drug-resistant A549-taxolR cells than in control A549 cells. Moreover, depletion of *hsf1* by the CRISPR/Cas9 system or stable transfection of shRNA to A549-taxolR significantly inhibited the enrichment of HSF1 in *mdr1* promoter region (Fig. 2e, Supplementary Fig. 2b). However, the CRISPR/Cas9 system to block the *hsf1* expression did not completely inhibit MDR function, suggesting partial involvement of HSF1 in MDR regulation. In the case of knockdown of *mdr1* (siMDR1) to A549-taxolR and T47D-doxR cells, HSF1 expression at both mRNA and protein levels was not altered (Supplementary Fig. 3a, b). Moreover, wild-type (WT) *mdr1* overexpression to control A549 and T47D cells did not affect *hsf1* expression (Supplementary Fig. 3c), suggesting HSF1 as an upstream molecule for MDR1 overexpression in drug-resistant cells.

### Phosphorylation of HSF1 at Ser303/307 involved in transcriptional activation of *mdr1*

**Fig. 3:**
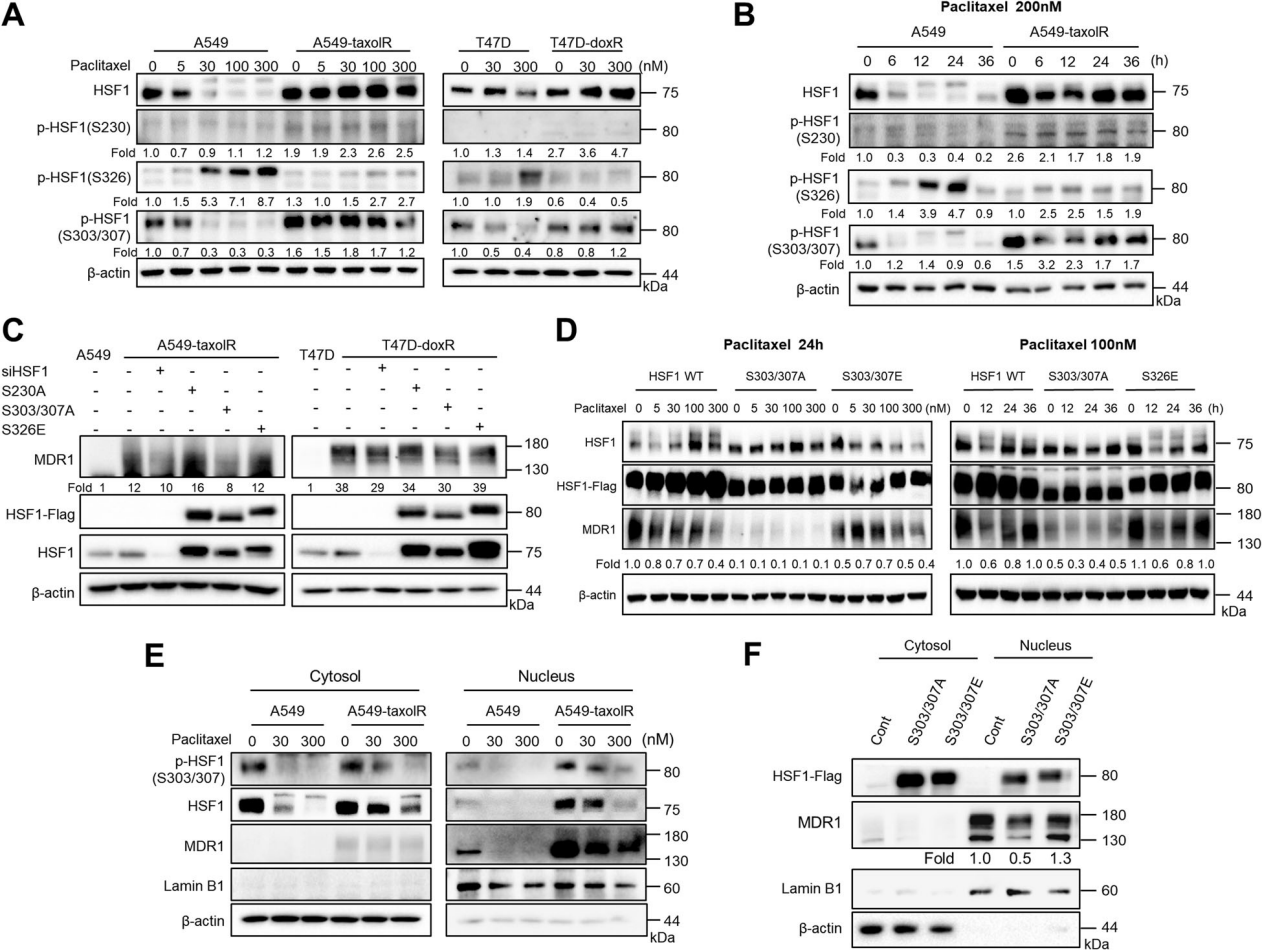
Phosphorylation patterns of HSF1 in drug-resistant cancer cells. **a**, **b** Protein levels in A549 lung cancer cells, paclitaxel-resistant A549 cells (A549-taxolR), T47D breast cancer cells, and doxorubicin-resistant T47D cells (T47D-doxR) after treatment for indicated times and with indicated concentrations of paclitaxel were examined by western blot analysis. **c** A549-taxolR cells and T47D-doxR cells were transiently transfected with FLAG-tagged point mutants of HSF1 at Ser230 (S230A, phospho-defective), Ser326 (S326E, phospho-mimicking), and Ser303/307 (S303/307A, phospho-defective), and western blotting was performed. **d**, left A549-taxolR cells after transfection with FLAG-tagged point mutants of HSF1 at Ser303/307 (S303/307A and S303/307E, phospho-defective and mimicking, respectively) were treated with indicated concentrations of paclitaxel for 24 h. **d**, right A549-taxolR cells after transfection with FLAG-tagged point mutants of HSF1 at Ser303/307 (S303/307A, phospho-defective) or Ser326 (S326E, phospho-mimicking) were treated paclitaxel for indicated times. **e** Western blotting was performed using cytosolic and nuclear fractions from A549 cells and A549-taxolR cells. Fraction purity and equal loading were assessed by western blots for lamin B1 and β-actin. **f** HEK293T cells after transfection with FLAG-tagged point mutants of HSF1 at Ser303/307 (S303/307A and S303/307E, phospho-defective and mimicking, respectively) were fractionated. Protein levels were quantified using Image J software, and data are expressed as the fold change relative to the untreated control.

Because *hsf1* mRNA level was not changed in drug-resistant cancer cells, post-translational modification of HSF1, such as phosphorylation patterns after paclitaxel treatment, was evaluated. Phosphorylation of Ser230 was slightly elevated and that of Ser326 was dramatically decreased in A549-taxolR cells when compared with control A549 cells. However, the phosphorylation of HSF1 at Ser303/307 was found to be dramatically increased in A549-taxolR cells, T47D-doxR cells, and MCF7-doxR cells. In control A549 and T47D cells, paclitaxel dose-dependent increase of HSF1 phosphorylation at Ser326 was found at 24 h of treatment; however, in drug-resistant cells, this increase was not found. HSF1 phosphorylation at Ser303/307 was inhibited by paclitaxel treatment in control cells; however, in drug-resistant cells, this inhibition was not found; paclitaxel did not affect the phosphorylation of HSF1 at Ser303/307 in drug-resistant cells (Fig. 3a, Supplementary Fig. 4a). Regarding other HSF1 phosphorylations, such as those at Ser216 or Ser419, these expressions were not evidently altered by 12 h paclitaxel treatment in drug-resistant cells (Supplementary Fig. 4b). HSF1 phosphorylation of Ser303/307 in A549-taxolR cells was slightly inhibited from 6 to 12 h of paclitaxel treatment; however, at 24 h of the treatment, it started recovering to a level comparable to that in paclitaxel untreated cells, whereas HSF1 phosphorylation of Ser303/307 in control cells was dramatically reduced from 6 h of paclitaxel treatment (Fig. 3b). Moreover, recovery after heat-shock treatment only affected the phosphorylation of HSF1 at Ser303/307 without any changes to phosphorylation sites of HSF1 at Ser230 and Ser326 in A549-taxolR cells (Supplementary Fig. 4c).

To examine direct correlation between the phosphorylation of HSF1 and MDR1 expression, A549-taxolR cells were transfected with HSF1 phosphorylation mutants. In the case of HSF1 at Ser303/307, both phospho-defective and phospho-active mutants (S303/307A and S303/307E, respectively) were used. Only the phospho-defective mutant S303/307A showed decreased MDR1 expression in both A549-taxolR and T47D-doxR cells. For mimicking unphosphorylated HSF1 at Ser230, S230A, the phospho-defective mutant form of HSF1, was transfected, and for mimicking phosphorylated HSF1 at Ser326, the phospho-active mutant S326E was transfected, and these mutants did not affect MDR1 expression (Fig. 3c). We also treated A549-taxolR cells with paclitaxel and found that S303/307A showed decreased expression of MDR1 in terms of mRNA and protein levels, whereas S303/307E showed increased MDR1 expression. Another mutant of S326 did not affect MDR1 expression despite paclitaxel treatment (Fig. 3d, Supplementary Fig. 4d). However, when the expression of HSP70 was examined using mimicking and defective mutants of Ser303/307, a defective mutant of Ser303/307A showed increased expression of HSP70, whereas Ser303/307E showed vice versa (Supplementary Fig. 4e), which has already been reported^32^. HSF1 and pHSF1 at Ser303/307 were located not only in the cytosol but also in the nuclear fractions in drug-resistant A549-taxolR cells, whereas in parent cells, they were mainly distributed in the cytosol. MDR1 localized mainly in the nucleus, and this was observed more in drug-resistant cells than in parent cells (Fig. 3e). When immunofluorescence staining for pHSF1 at Ser303/307 was performed, fluorescence intensity of pHSF1 at Ser303/307 in the nucleus was stronger in their drug-resistant A549-taxolR cells than in their parent A549 cells (Supplementary Fig. 5a). To elucidate whether the phosphorylation of Ser303/307 affects the nuclear localization of HSF1, S303/307A and S303/307E were transfected to HEK293T cells, and nuclear localization was examined. Even though the distribution of S303/307A and S303/307E was similar in the cytosol and nucleus, only S303/307A inhibited MDR1 expression in the nucleus (Fig. 3f).

### Down-regulation of FBXW7 in drug-resistant cells involved in the inhibition of ubiquitin degradation of phosphorylated HSF1 at Ser303/307

**Fig. 4:**
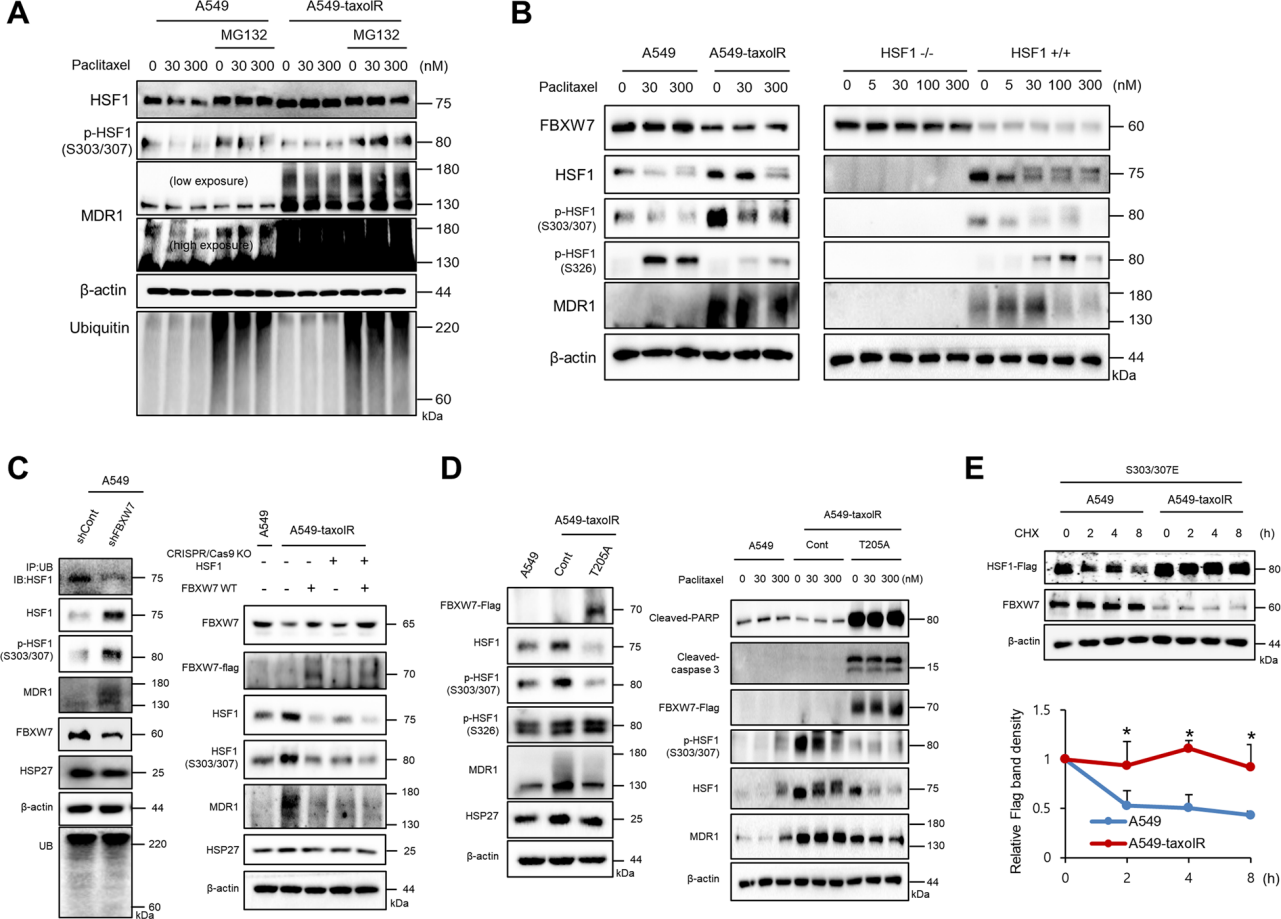
Decreased expression of FBXW7 in drug-resistant cells inhibited ubiquitin protein degradation of pHSF1 at Ser303/307. **a** A549 and A549-taxolR cells were treated with indicated concentrations of paclitaxel with or without MG132 (10 μM) for 12 h, and western blotting was performed. **b** Cells of A549, A549-taxolR, HSF1−/−, and HSF1+/+ mouse embryonic fibroblasts were treated with indicated concentrations of paclitaxel for 24 h, and western blotting was performed. **c**, left Immunoprecipitation (IP) was performed using A549 cells stably transfected of shFBXW7. **c**, right Western blotting was performed using A549 and A549-taxolR cells after transfection of HSF1 CRISPR/Cas9 KO or WT-FBXW7. **d** A549 and A549-taxolR cells were transiently transfected with point mutants of FBXW7 at Thr205 (T205A, phospho-defective), and western blotting was performed. **e** Western blot analysis in the phospho-mimicking mutant of HSF1 at Ser303/307 (S303/307E)-transfected A549-taxolR cells was performed after treatment with 10 μg/mL cycloheximide (CHX) for various time periods. Protein levels were quantified using Image J software, and data are expressed as the fold change relative to the control. Results are presented as the mean ± SD of at least three independent experiments. Statistics calculated based on a Student’s *t*-test, *p < 0.05.

**Fig. 5:**
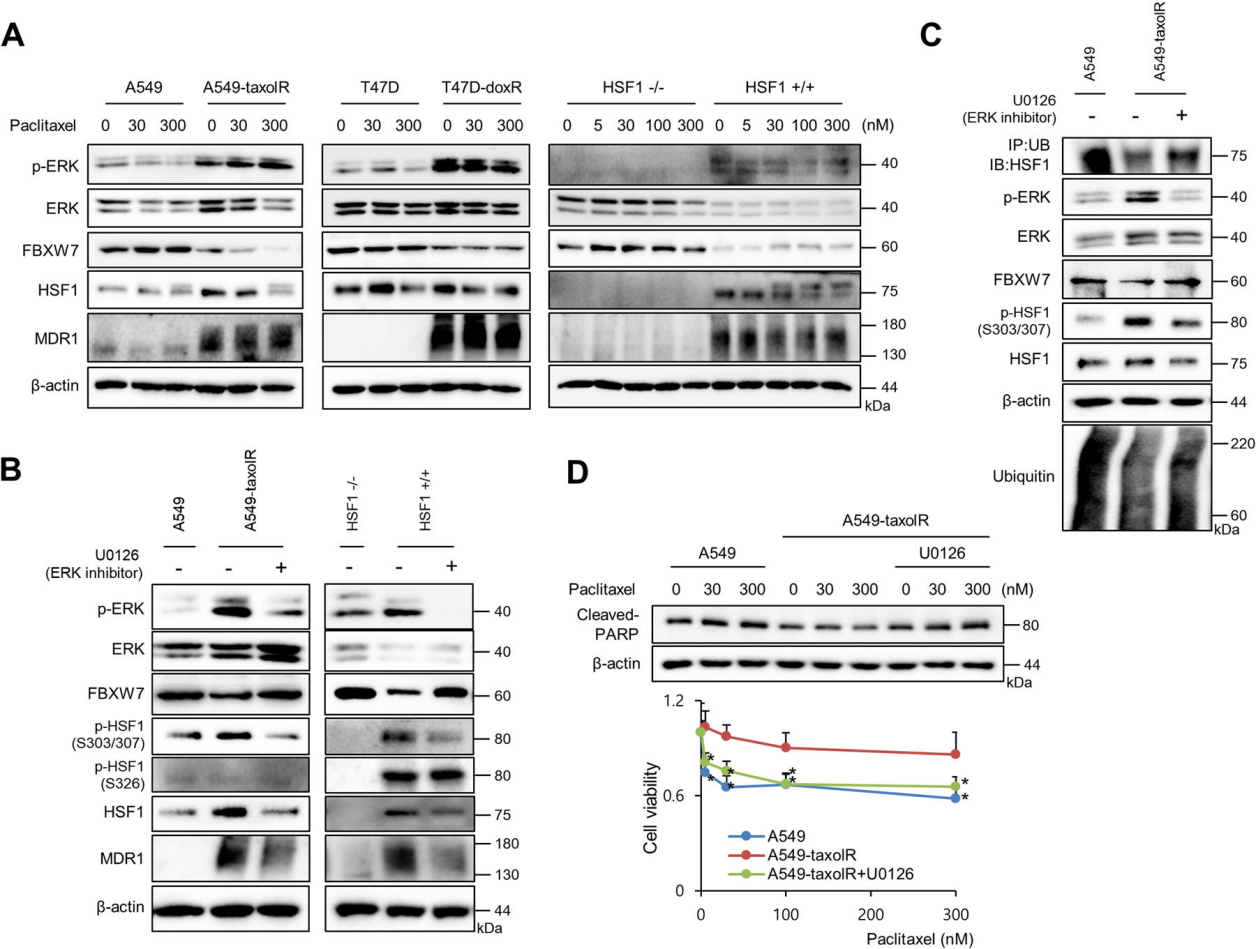
ERK1/2 activation in drug-resistant cancer cells was involved in decreased FBXW7 expression. **a** Cells of A549, A549-taxolR, T47D, T47D-doxR, HSF1−/− mouse embryonic fibroblasts, and HSF1+/+ mouse embryonic fibroblasts were treated with indicated concentrations of paclitaxel for 24 h, and western blotting was performed. **b** Cells of A549, A549-taxolR, HSF1−/− mouse embryonic fibroblasts, and HSF1+/+ mouse embryonic fibroblasts were treated with U0126, an ERK1/2 inhibitor (10 μM), for 12 h, and western blotting was performed. **c** Cell lysates of A549 and A549-taxolR with or without U0126 treatment (10 μM) for 12 h were immunoprecipitated with a ubiquitin construct (Ub) and immunoblotted with HSF1. Western blotting was also performed. **d** A549 and A549-taxolR cells were pretreated with or without U0126 treatment (10 μM) for 1 h and were treated with indicated doses of paclitaxel. Cell death was analyzed by western blot analysis, and cell viability was determined by the MTT assay. Values are presented as percentages of cell survival in paclitaxel-treated cells relative to untreated cells and as the mean ± SD of at least three independent experiments. Statistics calculated based on a Student’s *t*-test, *p < 0.05.

Because sustained phosphorylation of HSF1 at Ser303/307 was involved in MDR expression in drug-resistant cells, we examined the protein stability of HSF1 according to the phosphorylation status of Ser303/307. Treatment with MG132, a proteasome inhibitor, increased the stability of pHSF1 at Ser303/307 in A549 cells; however, no significant difference was seen in A549-taxolR cells (Fig. 4a). To elucidate the mechanisms involved in increased protein stability of pHSF1 at Ser303/307 in drug-resistant cells, upstream signaling was investigated; it was found that FBXW7, a ubiquitin E3 ligase which reportedly interacts with pHSF1 at Ser303/307 and induces HSF1 degradation^14^, was down-regulated in A549-taxolR cells and T47D-doxR cells even after paclitaxel treatment (Figs. 4b and 5a). However, the mRNA level of *fbxw7* was not altered (Supplementary Fig. 5b). Stably knockdowned *fbxw7* with shRNA FBXW7 to A549 cells increased pHSF1 at Ser303/307 and the stability of HSF1, which resulted in increased MDR1 protein expression. Overexpression of FBXW7 in A549-taxolR and HSF1+/+ MEF cells led to enhanced proteosomal degradation of HSF1 by its direct binding to pHSF1 at Ser303/307, which resulted in reduced MDR1 expression (Fig. 4c, Supplementary Fig. 5c). Because pFBXW7 at T205 is involved in FBXW7 degradation^33^, we prepared a phospho-defective mutant of T205 (T205A). The transfection of T205A resulted in decreased stability of HSF1 and pHSF1 at Ser303/307 as well as decreased MDR1 expression. However, the expression of pHSF1 at Ser326 was not affected by T205A transfection to A549-taxolR cells. Moreover, T205A transfection to A549-taxolR cells showed increased expression of cleaved caspase-3 and cleaved PARP1, even without paclitaxel treatment (Fig. 4d). Increased protein stability of S303/307E after CHX treatment was noted in A549-taxolR cells when compared with that in parent A549 cells (Fig. 4e). Transfection of shFBXW7, FBXW7-WT, or T205A did not alter HSP27 expression (Fig. 4c, d), suggesting that the protein degradation of pHSF1 at Ser303/307 by FBXW7 is independent of its transcriptional role for HSPs.

### Activation of ERK1/2 in drug-resistant cells involved in decreased expression of FBXW7

ERK1/2 activation was frequently observed in various drug-resistant cells^34–36^, and ERK1/2 activation induces FBXW7 phosphorylation at T205, which is involved in FBXW7 degradation^33^. FBXW7 deficiency or loss-of function confers resistance to chemotherapeutics, such as paclitaxel^37^. Moreover, FBXW7 increased ubiquitin degradation of pHSF1 at Ser303/307 (ref. ^14^). Indeed, A549-taxolR cells and T47D-doxR cells showed increased activation of ERK1/2 accompanied with down-regulation of the FBXW7 protein. When HSF1+/+ and HSF1−/− MEF cells were compared, similar effects were observed (Fig. 5a). To elucidate the involvement of ERK1/2 activation in the expression of FBXW7, the resistant cells were treated with U0126 (an ERK1/2 inhibitor); consequently, we noted restored FBXW7 expression accompanied with degradation of pHSF1 at Ser303/307, thus resulting in characteristics very similar to those of control A549 cells. In the case of the expression of pHSF1 at Ser326, no alteration by ERK1/2 inhibitor was observed. When HSF1+/+ and HSF1−/− MEF cells were compared, similar phenomena were observed (Fig. 5b). Moreover, on treatment of drug-resistant cells with ERK1/2 inhibitor, increased ubiquitination of HSF1 and decreased protein stability of pHSF1 at Ser303/307 were detected (Fig. 5c), with sensitization to paclitaxel, when cleavage of PARP1 and cell survival were detected (Fig. 5d).

### Phosphorylation of HSF1 at Ser303/307 decreased paclitaxel-mediated cell death

**Fig. 6:**
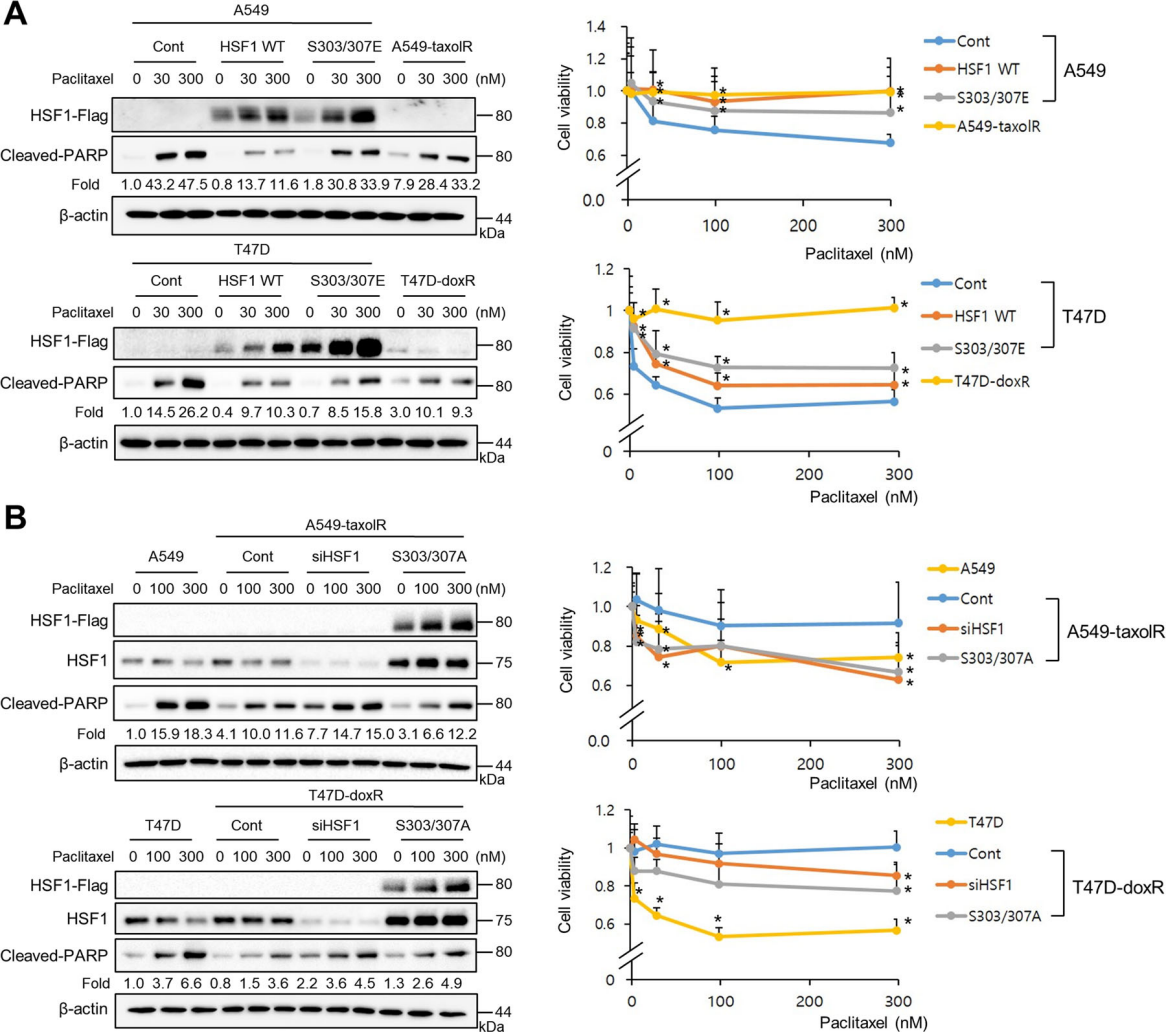
Increased phosphorylation of HSF1 at Ser303/307 in drug-resistant cancer cells was responsible for paclitaxel resistance. A549, A549-taxolR, T47D, and T47D-doxR cells after transient transfection of **a** WT-HSF1 and S303/307E, or **b** siRNA-HSF1 and S303/307A treated with indicated concentrations of paclitaxel for 24 h. Western blotting was performed. Cell viability was determined by the MTT assay. Data are expressed as the mean ± SD of at least three independent experiments. Protein levels were quantified using Image J software, and data are expressed as the fold change relative to the negative control. Statistics calculated based on a Student’s *t*-test, *p < 0.05 vs each control (Cont) of parent or resistant cells.

Because drug-resistant cells showed increased pHSF1 at Ser303/307 and increased MDR1 expression, the relationship between HSF1 phosphorylation of Ser303/307 and drug resistance was examined. The transfection of HSF1 WT or S303/307E to control A549 cells or T47D cells reduced PARP1 cleavage by paclitaxel treatment, and the levels were similar to those in A549-taxolR cells or T47D-doxR cells, respectively. However, the involvement of HSF1 in the development of paclitaxel resistance was partial in T47D-doxR, suggesting that HSF1 is more dominantly involved in drug resistance development of A549-taxolR than that of T47D-doxR. Moreover, the transfection of siHSF1 or S303/307A to A549-taxolR or T47D-doxR increased PAPR1 cleavage by paclitaxel treatment, and the levels were similar to those in A549 or T47D cells, respectively. In MTT assay, similar patterns were also shown (Fig. 6a, b).

### Correlation of pHSF1 at Ser303/307 with MDR1 expression in spontaneously induced rat mammary tumor tissues and human lung cancer tissues

**Fig. 7:**
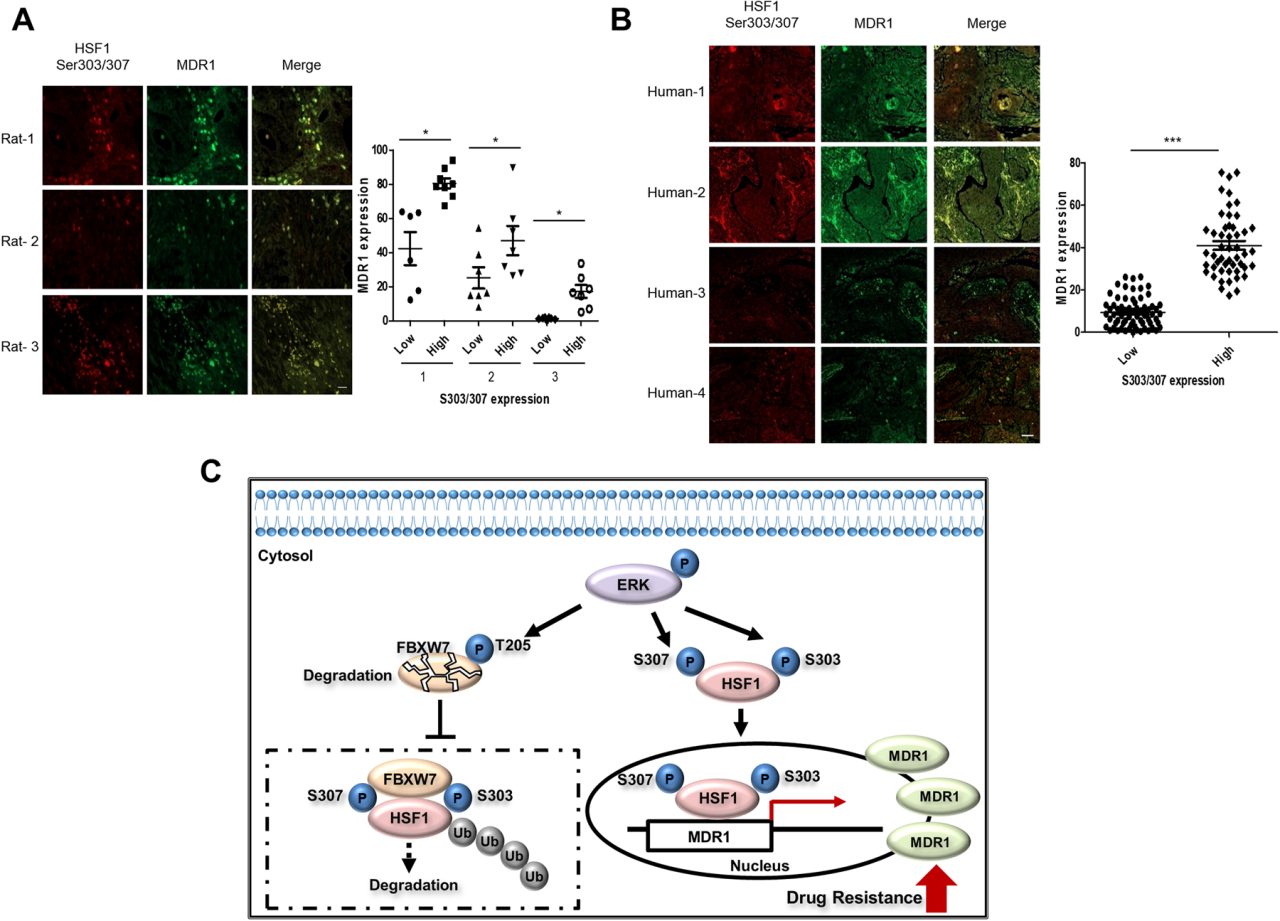
Positive correlation of pHSF1 at Ser303/307 and MDR1 in rat mammary tumors and human lung cancer tissues. Expression of pHSF1 at Ser303/307 and MDR1 expression were evaluated by immunofluorescence in rat mammary tumor tissues (total three tissues with adenocarcinomas) (**a**) and human lung cancer tissues (120 human lung cancer tissues with adenocarcinomas) (**b**) (Ser303/307: red; MDR1: green). Representative pictures of stained mammary tumor tissues (scale bar, 20 μm) (representative image of mammary tumors from three independent rats) and human lung cancer tissues (scale bar, 100 μm) (four representative images from 120 human lung cancer tissues) are presented. Quantification of Ser303/307-positive and MDR1-positive areas in each slide was analyzed using image analyzer (Image J). Statistics calculated based on one-way ANOVA, *p < 0.05, ***p < 0.005. **c** Hypothetical scheme of HSF1 phosphorylation at Ser303/307 for *mdr1* promoter activation in drug-resistant cells. Increased pERK1/2 in cancer drug-resistant cells induces FBXW7 phosphorylation at Thr205 and protein degradation of FBXW7, which results in increased protein stability of pHSF1 at Ser303/307. Phosphorylated HSF1 at Ser303/307 directly interacts with the promoter site of *mdr1* and increases its promoter activity, which is finally associated with drug resistance development in cancer cells.

To elucidate the relationship between MDR1 and pHSF1 at Ser303/307 in mammary tumors, expressions of MDR1 and pHSF1 at Ser303/307 were examined using DMBA-induced rat mammary tumors. All rat mammary tumors induced by DMBA were malignant adenocarcinomas. The immunofluorescence of pHSF1 at Ser303/307 (red) was co-localized in MDR1 expressed mammary tumors (green). Moreover, high expression of pHSF1 at Ser303/307 showed high levels of MDR1, and low expression of pHSF1 at Ser303/307 showed low level of MDR1, thus indicating a positive correlation (Fig. 7a). When human lung cancer tissue slides of 120 patient specimens were examined, similar expression patterns were observed (Fig. 7b), suggesting positive correlation between the expression of pHSF1 at Ser303/307 and MDR1.

## Discussion

In this study, HSF1 was identified as an important factor in the transcriptional activation of *mdr1*, which was finally identified to be involved in drug resistance. Drug-resistant cells of A549-taxolR, T47D-doxR, and MCF7-doxR showed overexpression of MDR1 at both mRNA and protein levels. Furthermore, the phosphorylation of HSF1 at Ser303/307, which are the characteristic post-translational modification sites of HSF1, plays an important role in the transcriptional activation of *mdr1*.

Paclitaxel- and doxorubicin-resistant cancer cells all showed resistance to paclitaxel and overexpression of *mdr1* at a transcriptional level, suggesting that the administration of different chemotherapeutic drugs increased *mdr1* gene and showed multidrug resistance. Interestingly, HSF1 was overexpressed, and paclitaxel treatment did not degrade HSF1 protein in drug-resistant cancer cells unlike in parent cells; this phenomenon was more dominant in paclitaxel-resistant cells, such as A549-taxolR cells, than in doxorubicin-resistant cells, such as T47D-doxR cells. Since the mRNA level of *hsf1* was not altered, post-translational modification, such as phosphorylation, was examined, and it was found that pHSF1 at Ser303/307 was not degraded in drug-resistant cells. Regarding other phosphorylations of HSF1, such as those at Ser326 and Ser230, MDR1 expression was not related. The phosphorylation of HSF1 at Ser303/307 normally appears to repress the transactivation capacity of HSF1 for HSP transcription^38^ and is involved in cytoplasmic localization of HSF1 (ref. ^39^). Indeed, the phospho-mimicking mutant S303/307E showed inhibited expression of HSP70 at both mRNA and protein levels; however, MDR1 expression was found to be increased. However, the phospho-defective mutant S303/307A showed the opposite trend, suggesting that pHSF1 at Ser303/307 acts as a partly inhibitory transcriptional factor of HSP expression under normal physiological growth conditions, whereas in the case of regulation of *mdr1* transcription during the drug resistance development process by continuous treatment with anticancer drugs, it acts as a transcriptional activator of *mdr1* expression. Since the *mdr1* gene has an HSE and it has been reported that cells overexpressing WT-HSF1 show increased expression of ABC transporters, including MDR1/ABCB1, and deletion of the HSF1 protein residues 221–315, which includes HSF1 phosphorylation sites S303/307, did not increase MDR1 expression^40^, the phosphorylation of Ser303/307 may also have transactivating activity. Moreover, there are evidences of predominant nuclear localization of pHSF1 at Ser303/307 (ref. ^41^), and our data also suggested predominant nuclear localization of pHSF1 at Ser303/307. In this study, the activated form of HSF1 (pHSF1 at Ser326 and S230) for HSP induction did not exhibit the regulation of MDR1. Since the regulation of MDR1 is extremely complex and involves the interplay of various transcription factors depending on the circumstances of cells, the pHSF1 at Ser303/307 may interact with other transcription factors for *mdr1* transcription. However, this complexity and diversity in the regulation of MDR1 may add to the difficulty in finding the exact interplay with other factors.

Among the few papers that have been published on the relationship between HSF1 and MDR1, some suggested positive correlation between MDR1 and HSF1, wherein HSF1 up-regulated the expression of MDR1^4^. However, other papers suggested that HSF1 down-regulated the expression of MDR1. The difference between the two systems was that positive regulation was examined using mice or human cancer cells and negative regulation was examined using non-cancer tissues of mice, such as cardiomyocytes and liver tissues. Mouse genomes contain two paralogous genes, *mdr1a* and *mdr1b*, for P-glycoprotein, whereas humans have one *mdr1* gene^42^. The tissue distribution of *mdr1a/1b* in mice is broad, including the liver and heart; however, the *mdr1* gene of humans is expressed at relatively low levels in normal conditions, and a stressed condition, such as treatment with chemotherapeutic drugs targeting cancer cells, can overexpress the *mdr1* gene^28^. These different situations may differently regulate *mdr1* gene expression by HSF1.

The up-regulation of pHSF1 at Ser303/307 was reportedly degraded by the FBXW7 ubiquitin ligase, and our drug-resistant cells showed decreased FBXW7 expression. Because FBXW7 degraded pHSF1 at Ser303/307 via ubiquitin degradation pathways, decreased expression of FBXW7 in drug-resistant cells may involve the inhibition of pHSF1 degradation. Indeed, knockdown of FBXW7 to parent cells or overexpression of FBXW7 to drug-resistant cells regulated pHSF1 at Ser303/307, while FBXW7 did not affect the phosphorylated form of Ser326, suggesting that the decreased expression of FBXW7 in drug-resistant cells specifically stabilized the pHSF1 at Ser303/307.

The phosphorylation of FBXW7 at T205 reportedly decreases FBXW7 protein stability^43^, and this phosphorylation was induced by ERK1/2 activation^33^. Drug-resistant cells showed activation of ERK1/2 (refs. ^34–36^) unlike their parent cells, which is key regulation of low expression of FBXW7 in drug-resistant cells. Indeed, treatment of ERK1/2 inhibitor to drug-resistant cells or phospho-defective mutants of FBXW7 at T205 (T205A) increased FBXW7 protein stability and degradation of pHSF1 at Ser303/307, which finally resulted in decreased expression of MDR1. These results suggested that ERK1/2 activation in drug-resistant cells phosphorylated FBXW7 at T205 and induced ubiquitin degradation of FBXW7, which inhibited the degradation of pHSF1 at Ser303/307 and promoted transcriptional activation of the *mdr1* gene.

Tissues from mammary tumors that were spontaneously induced by DMBA or from human lung cancers showed a co-localization of and positive correlation between pHSF1 at Ser303/307 and MDR1, suggesting the possibility that targeting pHSF1 at Ser303/307 will overcome MDR1-mediated drug resistance in cancer therapy (Fig. 7c).

Taken together, we show that decreased expression of FBXW7 in drug-resistant cells, which is mediated by ERK1/2 activation, specifically stabilized the pHSF1 at Ser303/307. Increased protein stability of pHSF1 promoted *mdr1* transcription by its direct interaction with the promoter site of *mdr1* in drug-resistant cells, providing, for the first time, the post-translational modification of HSF1 for MDR1 regulation during drug resistance development.

## Materials and methods

### Cell culture and treatments

A549 human lung cancer cells, paclitaxel-resistant A549 cells (A549-taxolR), T47D human breast cancer cells, doxorubicin-resistant T47D cells (T47D-doxR), MCF7 human breast adenocarcinoma cells, and doxorubicin-resistant MCF7 cells (MCF7-doxR) were maintained in RPMI-1640 medium and supplemented with 10% fetal bovine serum at 37 °C in an incubator with a humidified atmosphere of 95% air and 5% CO_2_. To maintain the paclitaxel resistance phenotype, A549-taxolR cells were maintained with occasional addition of 100 nM paclitaxel (Santa Cruz Biotechnology). Paclitaxel-resistant A549-taxol cells were kindly provided by Prof. S.K. Lee (Seoul National University, Seoul, South Korea). Doxorubicin-resistant T47D cells and doxorubicin-resistant MCF7 cells were kindly provided by Prof. Y.J. Kwon (Ewha Womans University, Seoul, South Korea). Wild type and *hsf1* knockout mouse embryonic fibroblast (HSF1+/+ and HSF1−/− MEF) cells were provided by Dr. Ivor J. Benjamin (University of Utah, Salt Lake City, UT). MEF (HSF1+/+ and HSF1−/− MEF) cells were cultured in Dulbecco’s minimal essential medium (DMEM) supplemented with heat-inactivated 10% fetal bovine serum (FBS) and antibiotics. Cell lines were tested by BioMycoX Mycoplasma PCR Detection Kit (JCBIO Co., Ltd) to ensure that they were mycoplasma-free. MG132 were purchased from Sigma Aldrich (St. Louis, MO, USA). A549 and A549-taxolR cells were treated with indicated concentrations of paclitaxel with or without MG132 (10 μM) for 12 h.

### Cell transfection

Wild-type (WT) human HSF1 was cloned into p3XFLAG-Myc-CMV containing an N-terminal FLAG-tag. The phosphorylation mutant HSF1 (S230A, S326E, S303/307A, and S303/307E) constructs were constructed using overlapped extension primers. Plasmids used in this study are listed in Supplementary Table S1. Pre-designed siRNA for human HSF1 (sc-35611) and a negative control siRNA (30 nM) were purchased from Santa Cruz Biotechnology (Dallas, TX, USA). Pre-designed siRNA for *mdr1* was purchased from Bioneer (Daejeon, Korea). Transient transfection was carried out using Lipofectamine 2000 (Invitrogen), according to the manufacturer’s guidelines. HSF1 shRNA (sc-35611-V), FBXW7 shRNA (sc-37547-V), polybrene (sc-134220), and sh-control plasmid (sc-10808) were obtained from Santa Cruz Biotechnology. To produce sh-control cells and sh-HSF1 cells, cell lines were selected using puromycin (1 μg/mL) for at least 1 week. Control CRISPR/Cas9 (Cont) plasmid (sc-418922) and HSF1 CRISPR/Cas9 knockout (KO) plasmid (sc-400432-KO-2) were purchased from Santa Cruz Biotechnology. For CRISPR/Cas9 KO system transfection, cells were seeded and transfected after 24 h using plasmid transfection medium (sc-108062) containing transfection reagent (sc-395739).

### MTT [3-(4,5-dimethylthiazol-2-yl)-2,5-diphenyltetrazolium bromide] assay

Cell viability against paclitaxel-induced toxicity was determined using an MTT assay (Amersham Pharmacia Biotech) in 96-well plates. A549/A549-taxolR cells were seeded at a density of 2 × 10^4^ cells/well in 96-well plates and treated with the desired concentration of paclitaxel (0, 5, 30, 100, and 300 nM) for 24 h. The statistical significance was determined by the Student’s *t*-test. The differences were considered significant if the *p* value was less than 0.05.

### *mdr1* promoter assay

The MDR1-Luc plasmid was purchased from Addgene (Cambridge, MA, USA). *mdr1* promoter activity was measured using a luciferase assay system kit (#E4030), which was purchased from Promega (Madison, WI, USA). Protein was quantitated using a protein assay reagent (#500-0006), which was purchased from Bio-Rad (Hercules, CA, USA).

### Cycloheximide chase assay

FLAG stability was measured in the presence of cycloheximide (CHX, Sigma Aldrich) after transfection of S303/307A or S303/307E. At 24 h after transfection, the cells were split into multiple dishes, CHX was added (10 μg/mL), and the cells were harvested at the indicated times.

### Cell fractionation

Cellular fractionation was performed using a subcellular protein fractionation kit (#78840) purchased from Thermo Fisher Scientific (Waltham, MA, USA). The harvested cell pellets were resuspended in cytoplasmic extraction buffer and incubated at 4 °C for 10 min with gentle mixing. Samples were agitated every 5 min and then centrifuged at 500*g* for 5 min to collect the cytoplasmic fraction. Pellets were resuspended, incubated in nuclear extraction buffer at 4 °C for 30 min, and centrifuged at 5000*g* for 5 min to obtain the nuclear fraction.

### ChIP assay

A549 and A549-taxolR cells were fixed, nuclei were isolated, and chromatin was sheared by sonication using the ChIP Kit according to the manufacturer’s instructions (ab500; Abcam). The sheared chromatin was immunoprecipitated using HSF1 antibody (ab-52757; Abcam) and IgG (ab500; Abcam). Details regarding the primers used in ChIP-qPCR can be found in Supplementary Table S2. The quantitative PCR conditions were as follows: 10 min at 95 °C, followed by 38 cycles of denaturation (5 s at 95 °C), annealing (10 s at 62 °C), and extension (20 s at 72 °C) with single acquisition of fluorescence at the end of each extension step. ChIP-qPCR was calculated as follows: fold enrichment = 2^−((Ct IP)−(Ct mock))^. Mean values of three biological replicates were calculated. Statistical analysis was carried out using one-way ANOVA.

### Immunoblotting and immunoprecipitation

For Immunoblotting, A549/A549-taxolR cells were seeded at a density of 3 × 10^5^ cells/dish in 60-mm cell culture dishes. After 24 h incubation, cells were treated with paclitaxel varying from 5 to 300 nM. Cells were harvested at certain time points (6, 12, and 24 h). For immunoprecipitation, cells (1 × 10^6^) were lysed in immunoprecipitation buffer (50 mM HEPES, pH 7.6, 150 mM NaCl, 5 mM EDTA, 0.1% Nonidet P-40). After centrifugation (30 min at 15,000*g*) to remove particulate material, supernatants were incubated with antibodies (1:50) against ubiquitin with constant agitation at 4 °C. Immunocomplexes were precipitated with protein A/G PLUS-agarose (sc-2003; Santa Cruz Biotechnology) and analyzed by sodium dodecyl sulfate-polyacrylamide gel electrophoresis. Immunoblotting and immunoprecipitation were performed using the following antibodies: β-actin (sc-47778), HSP27 (sc-1048), pHSF1 (Ser230; sc-30443-R), p-ERK (sc-7383), Lamin B1 (sc-374015), and ubiquitin (sc-8017) were purchased from Santa Cruz Biotechnology. HSF1 (ab-52757), pHSF1 (Ser326; ab-76076), pHSF1 (Ser303/307; ab-81281), HSP70 (ab-1428), and FBXW7 (ab-109617) were purchased from Abcam (Cambridge, MA, USA). FLAG (#F3165) was purchased from Sigma Aldrich. MDR1 (#12683) and ERK (#9101) were purchased from Cell Signaling (Danvers, MA, USA). Protein band intensity was visualized on ChemiDoc (Bio-Rad) and quantified using Image J software 1.45 (National Institutes of Health, Bethesda, MD, USA).

### RT-PCR

Total RNA was extracted using QUIazol (Quiazen), and cDNA was synthesized using the ReverTra Ace RT-PCR Kit (Toyobo). *mdr1* and *gapdh* transcript levels were measured by RT-PCR (GenDEPOT). GAPDH was used as an internal control gene. The detailed primer sequences for RT-PCR are provided in Supplementary Table S3.

### Immunofluorescent staining

For cell immunofluorescence assays, cells were fixed with 10% paraformaldehyde, permeabilized with 0.1% Triton X-100 in phosphate-buffered saline (PBS), washed three times with PBS, and incubated with anti-HSF1 (Ser303/307) and anti-FLAG antibodies diluted 1:200 in PBS with 1% FBS overnight at 4 °C. The cells were incubated with Alexa 568-labeled anti-rabbit (1:500) and Alexa 488-labeled anti-mouse (1:500) secondary antibodies. After they were washed three times with PBS, coverslips were mounted onto microscope slides using a mounting reagent (Southernbiotech, Birmingham, AL, USA). The slides were then analyzed using a ZEISS LSM 880 Confocal Laser Scanning Microscope (Carl Zeiss).

For breast tissue immunofluorescence assays, spontaneous mammary tumors were induced in female Sprague–Dawley (SD) rats by oral administration of DMBA (15 mg per rat; Sigma Aldrich). Rats were autopsied under anesthesia at 26 weeks after DMBA administration. Detailed experimental procedures have been published previously^44^. All animals were randomized but not performed blind experiments. Human lung cancer tissue slides were purchased from US Biomax. For antigen retrieval, slides were placed in citric acid buffer (pH 6.0) and heated at 100 °C for 20 min. Slides were co-immunostained with HSF1 (Ser303/307) (1:200) and MDR1 (1:200) overnight at 4 °C. The slides were incubated with Alexa 568-labeled anti-rabbit (1:500) and Alexa 488-labeled anti-mouse (1:500) secondary antibodies. The nucleus was counterstained with DAPI (Sigma Aldrich), and the stained cells were imaged using a Zeiss Apotome (Carl Zeiss). Quantification of images was measured with image analyzer (Image J, NIH, Bethesda, MD, USA). All statistical analyses of images were performed using GraphPad Prism software 5.0 (GraphPad Software, San Diego, CA, USA).

### Statistical analysis

Data expressed as mean ± SD represented at least three independent experiments. Statistical significance was determined by Student’s *t*-test or one-way ANOVA (Newman–Keuls test). The differences were considered significant if the *p* value was <0.05. ANOVA test was performed using GraphPad Prism software 5.0.

## Supplementary information

Supplementary Legends

Supplementary Figure 1

Supplementary Figure 2

Supplementary Figure 3

Supplementary Figure 4

Supplementary Figure 5

## Data Availability

All data generated or analyzed during this study are included in this published article and its supplementary information files.
