# Supplementary material for: Decreased expression of FBXW7 by ERK1/2 activation in drug-resistant cancer cells confers transcriptional activation of MDR1 by suppression of ubiquitin degradation of HSF1
Source: Cell Death Dis. 2020 May 26;11(5):395. doi: 10.1038/s41419-020-2600-3 (PMC7251134; doi:10.1038/s41419-020-2600-3)
Supplement: Supplementary file 1 — Supplementary Legends [file 41419_2020_2600_MOESM1_ESM.docx]

**Supplemental Figure Legends**

**Supplementary Figure 1. Cell death by paclitaxel treatment in drug resistant cancer cells.** A549 lung cancer cells, paclitaxel-resistant A549 cells (A549-taxolR), T47D breast cancer cells, doxorubicin-resistant T47D cells (T47D-doxR), MCF7 breast adenocarcinoma cells, doxorubicin-resistant MCF7 cells (MCF7-doxR) were treated paclitaxel for 24 h at indicated concentrations. Cell death was analyzed by (**a**) Western blot analysis and cell viability was determined by the (**b**) MTT assay. Values are presented as percentages of cell survival in paclitaxel-treated cells relative to untreated cells and as mean ± SD of at least three independent experiments. Statistics calculated based on a student’s t-test, **p* < 0.05.

**Supplementary Figure 2. HSF1 depletion downregulated the transcriptional level of MDR1 in drug resistant cancer cells.** (**a** left) After A549 and A549-taxolR cells were stably transfected with shRNA (shCont) or shHSF1 and RT-PCR was performed. (**a** right) Luciferase assays in A549 and A549-taxolR cells were performed after transfection with a luciferase reporter construct with the mdr1 promoter with or without stably transfection of shRNA of HSF1. Values are expressed as fold-change relative to control A549 cells. Values are presented as mean ± SD of at least three independent experiments. Statistics calculated based on a student’s t-test, **p* < 0.05. **b** A549 and A549-taxolR cell line stably transfected with shCont or shHSF1 analyzed by ChIP assay to measure enrichment of MDR1 promoter sequences. IgG was used as a negative control for the HSF1 antibody. Statistics calculated based on one-way ANOVA, **p* < 0.05.

**Supplementary Figure 3. Effect of MDR1 depletion on HSF1 expression in drug resistant cancer cells.** A549 lung cancer cells, paclitaxel-resistant A549 cells (A549-taxolR), T47D breast cancer cells, and doxorubicin-resistant T47D cells (T47D-doxR) were transfected with a control siRNA (siCont), siMDR1, Cont-vector or MDR1 WT. Cell lysate was performed by Western blotting (**b**) or RT-PCR (**a**, **c**).

**Supplementary Figure 4. Phosphorylation patterns of HSF1 in drug resistant cancer cells. a** A549 cells, A549-taxolR, T47D cells, T47D-doxR, MCF7 cells, and MCF7-doxR were treated paclitaxel for 24 h at indicated concentrations. **b** Protein levels in A549 cells and A549-taxolR cells after treatment of indicated time and concentrations of paclitaxel, were examined by Western blot analysis. **c** Recovery after heat shock treatment were evaluated changes of phosphorylation sites of HSF1 at Ser230, Ser326 and Ser303/307 in A549-taxolR cells. **d** RT-PCR analysis in S303/307A and siHSF1 transfected A549-taxolR or T47D-doxR cells were performed. **e** A549 cells and A549-taxolR cells after transfection of Flag tagged point mutants of HSF1 at Ser303/307 (S303/307A and S303/307E, phospho-defective and mimicking, respectively) analyzed by Western blot.

**Supplementary Figure 5. FBXW7 mRNA level in resistance cells. a** A549 cells and A549-taxolR cells were treated paclitaxel for 24 h and stained with pHSF1 (Ser303/307) antibody and DAPI. Cells were analyzed using Confocal Laser Scanning Microscope. Scale bar, 40 μm. **b** RT-PCR using A549 cells, A549-taxolR cells, T47D cells, and T47D-doxR were performed. The *gapdh* was used as a loading control for RT-PCR. **c** Western blotting was performed using HSF1−/− mouse embryonic fibroblasts and HSF1+/+ mouse embryonic fibroblasts after transiently transfection of WT-FBXW7.

**Supplemental Tables**

**Supplementary Table 1. Plasmids used in this work**

| Name | | Insert | Vector | Marker |
| --- | --- | --- | --- | --- |
| HSF1 | Wild Type | Full length | p3xFLAG-Myc-CMV-26 | ampicillin |
|  | S303/307A | Point mutation | p3xFLAG-Myc-CMV-26 | ampicillin |
|  | S303/307E | Point mutation | p3xFLAG-Myc-CMV-26 | ampicillin |
|  | S230A | Point mutation | p3xFLAG-Myc-CMV-26 | ampicillin |
|  | S326E | Point mutation | p3xFLAG-Myc-CMV-26 | ampicillin |
| FBXW7 | Wild Type | Full length | p3xFLAG-Myc-CMV-26 | ampicillin |
|  | T205A | Point mutation | p3xFLAG-Myc-CMV-26 | ampicillin |

**Supplementary Table 2. Primer sets used in ChIP-qPCR**

| Gene symbols | Primer sequences (5’-3’) |  |
| --- | --- | --- |
|  |  |  |
| *mdr1* | GGA GCA GTC ATC TGT GGT GA | F |
|  | CTC GAA TGA GCT CAG GCT TC | R |

**Supplementary Table 3. Primer sequences for RT-PCR**

| Gene symbols | Primer sequences (5’-3’) |  |
| --- | --- | --- |
|  |  |  |
| *mdr1* | AGG AGG CCA ACA TAC ATG CC | F |
|  | AGA GTT CAC TGG CGC TTT GT | R |
| *hsf1* | GGT CAA GCC AGA GAG AGA CG | F |
|  | CTC ATG CTT CAT GGC CAG GA | R |
| *fbxw7* | AAA GAG TTG TTA GCG GTT CTC G | F |
|  | CCA CAT GGA TAC CAT CAA ACT G | R |
| *hsp27* | CCT GGA TGT CAA CCA CTT CG | F |
|  | CTG GGA TGG TGA TCT CGT TG | R |
| *hsp70* | ACA AGT CCG AGA ACG TGC AG | F |
|  | GCA TCG ATG TCG AAG GTC AC | R |
| *gapdh* | ACG GAT TTG GTC GTA TTG GG | F |
|  | TGA TTT TGG AGG GAT CTC GC | R |

F, forward; R, reverse.
