# Supplementary figures and images for: Decreased expression of FBXW7 by ERK1/2 activation in drug-resistant cancer cells confers transcriptional activation of MDR1 by suppression of ubiquitin degradation of HSF1
Source: Cell Death Dis. 2020 May 26;11(5):395. doi: 10.1038/s41419-020-2600-3 (PMC7251134; doi:10.1038/s41419-020-2600-3)

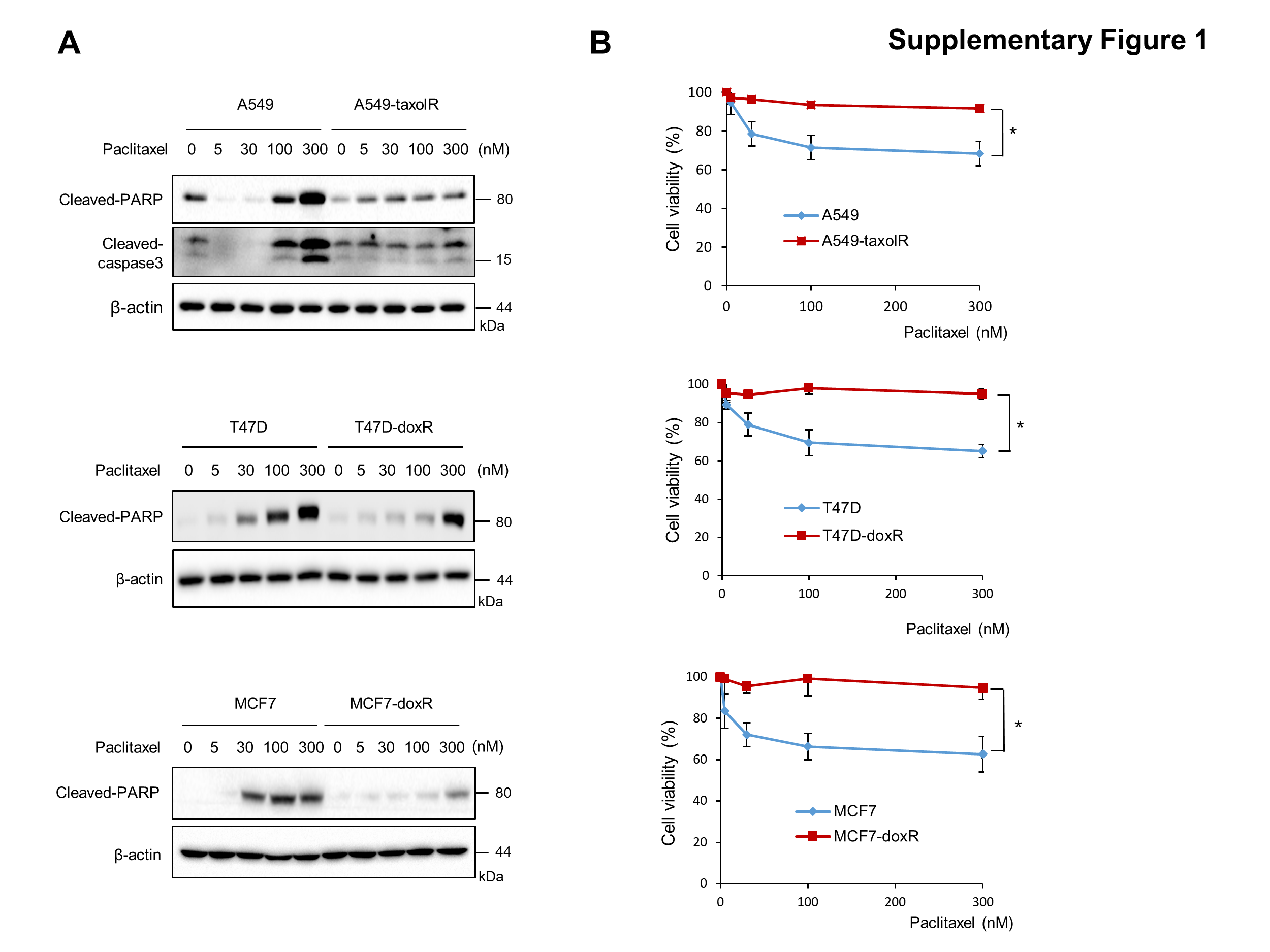

Supplement: Supplementary file 2 — Supplementary Figure 1 [file 41419_2020_2600_MOESM2_ESM.tif]

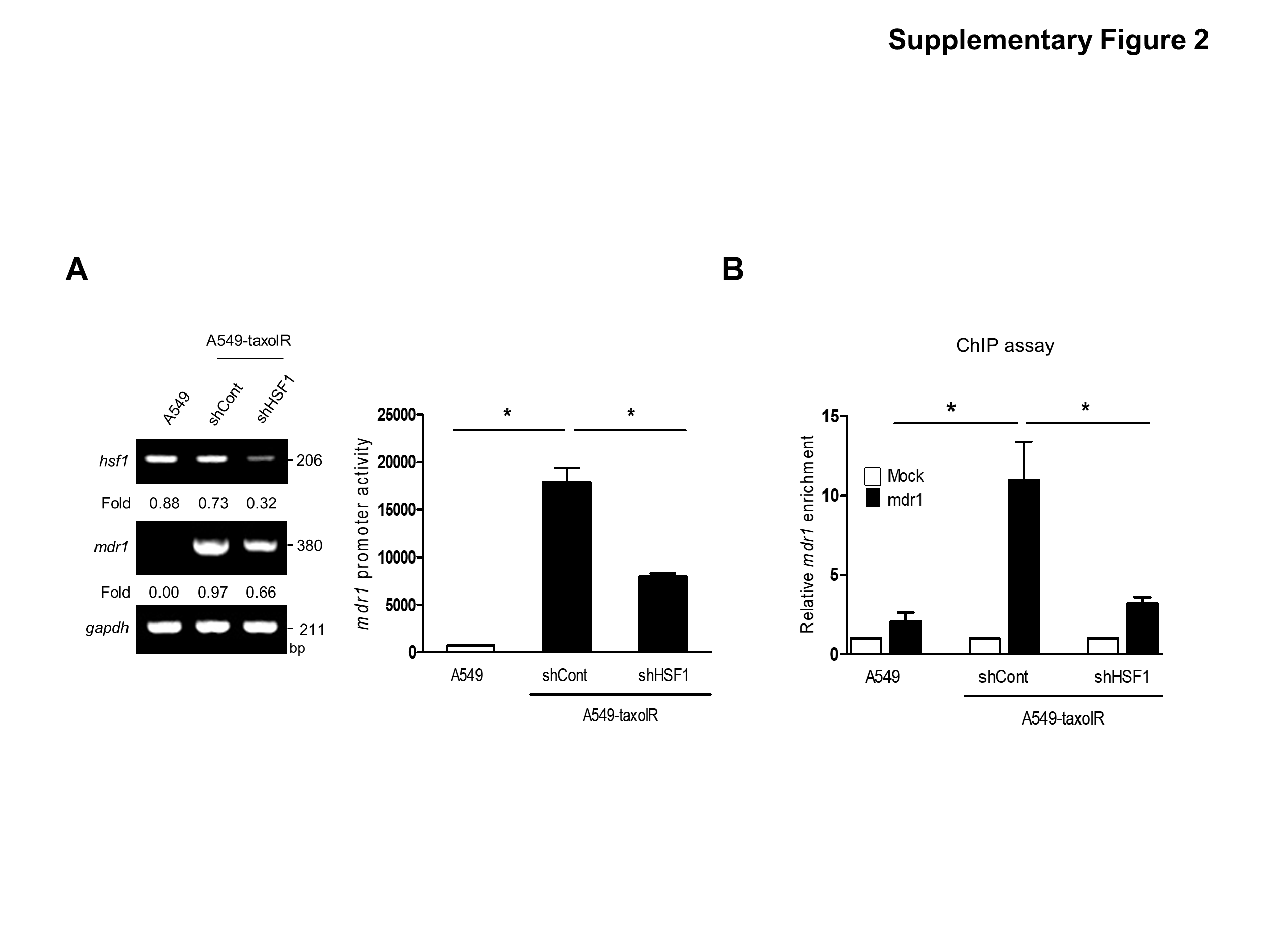

Supplement: Supplementary file 3 — Supplementary Figure 2 [file 41419_2020_2600_MOESM3_ESM.tif]

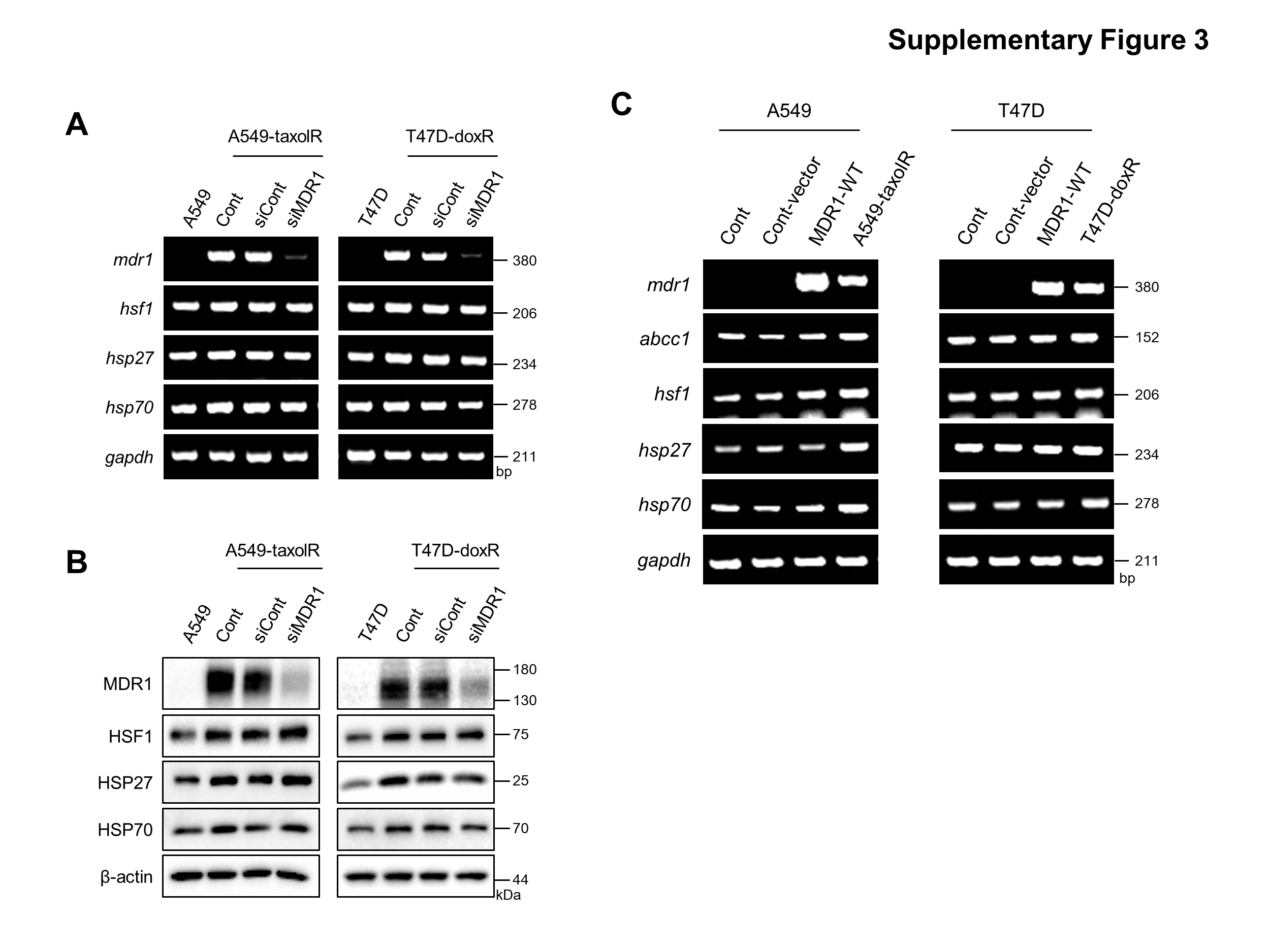

Supplement: Supplementary file 4 — Supplementary Figure 3 [file 41419_2020_2600_MOESM4_ESM.tif]

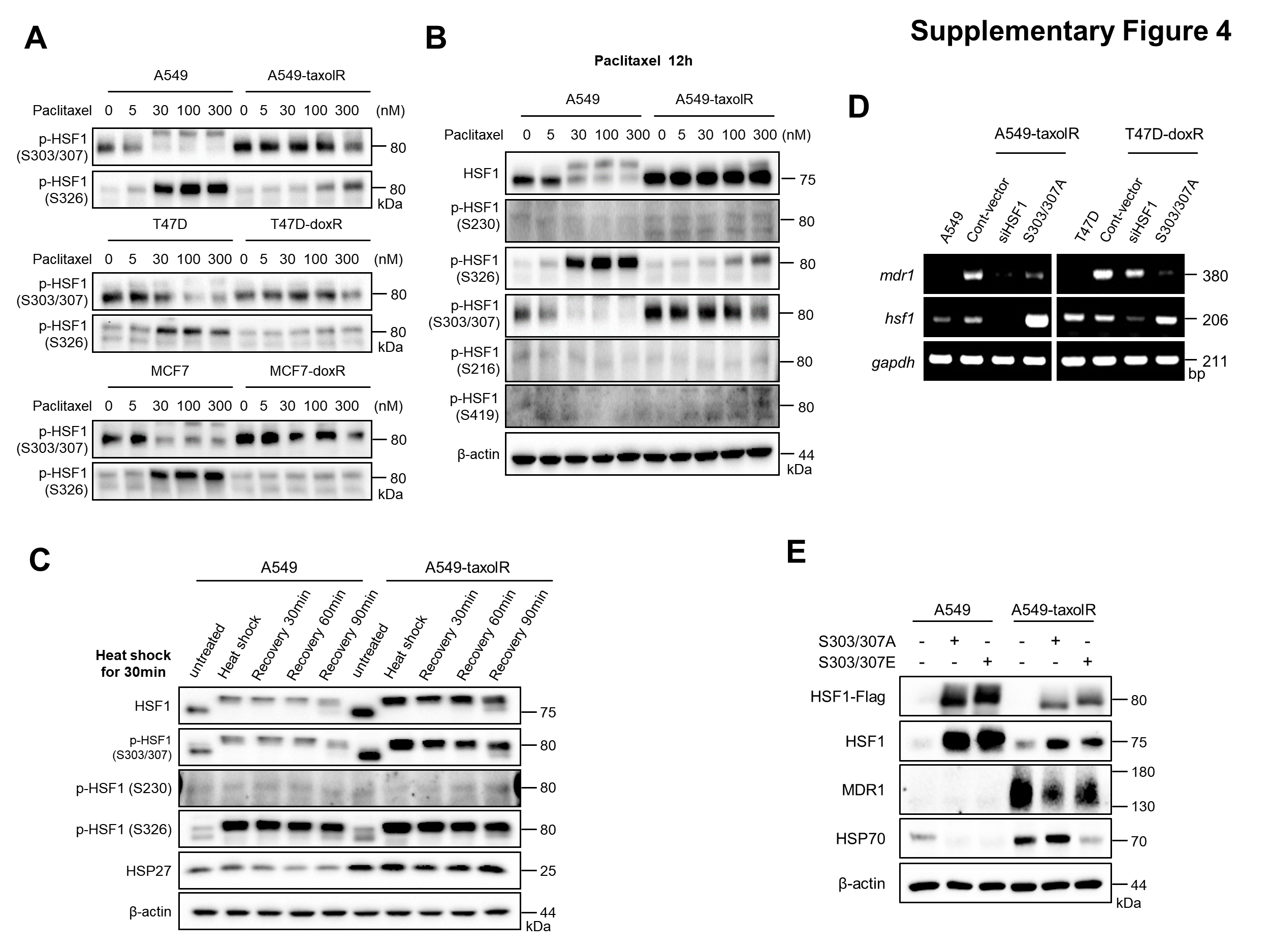

Supplement: Supplementary file 5 — Supplementary Figure 4 [file 41419_2020_2600_MOESM5_ESM.tif]

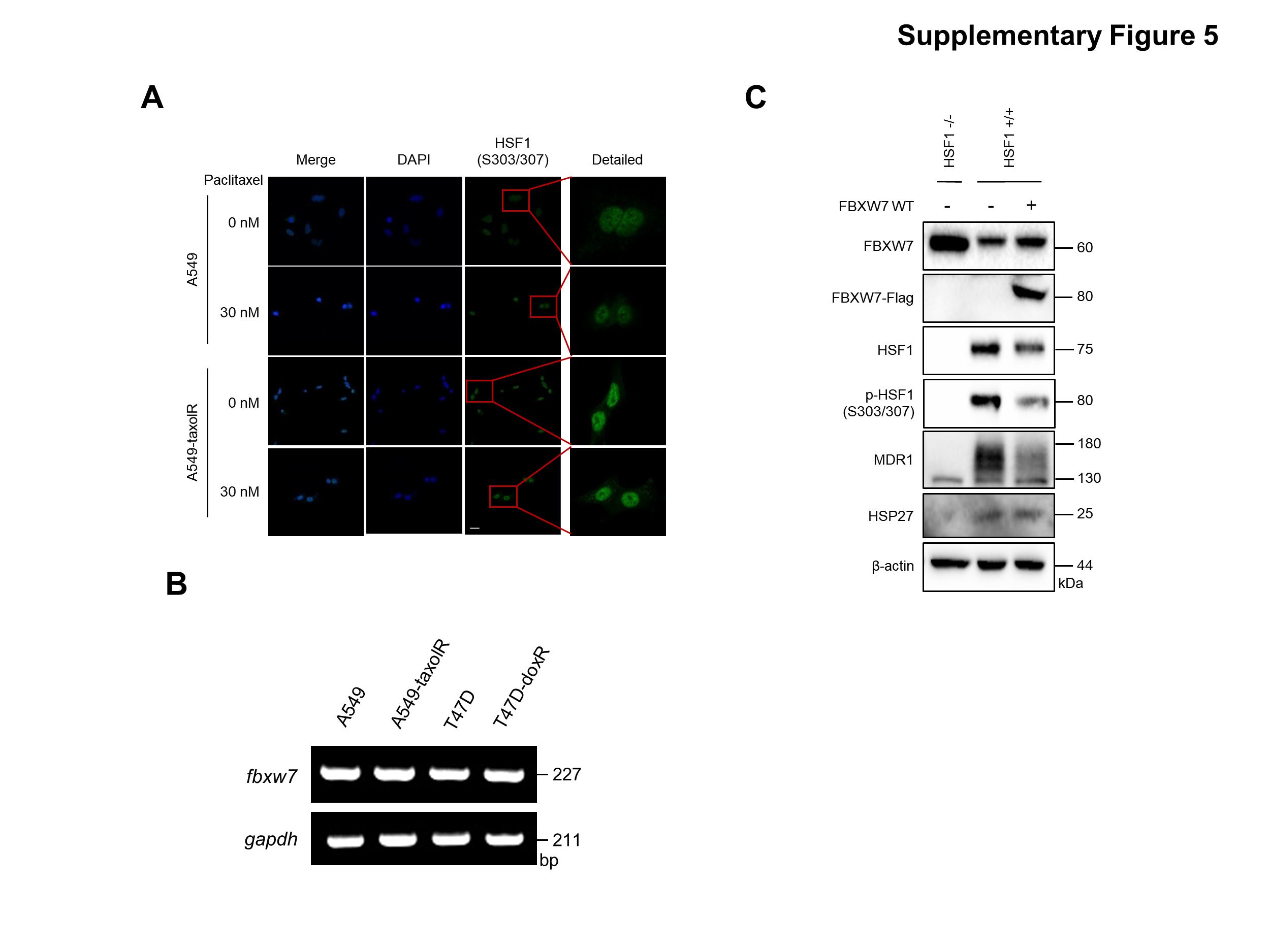

Supplement: Supplementary file 6 — Supplementary Figure 5 [file 41419_2020_2600_MOESM6_ESM.tif]
